# Colorectal Cancer and Bone Tissue: Fantastic Relations and Where to Find Them

**DOI:** 10.3390/cancers12082029

**Published:** 2020-07-24

**Authors:** Isabella Gigante, Valeria Tutino, Valentina De Nunzio, Maria Notarnicola

**Affiliations:** Laboratory of Nutritional Biochemistry, National Institute of Gastroenterology “S. de Bellis” Research Hospital, 70013 Castellana Grotte, Italy; valeria.tutino@irccsdebellis.it (V.T.); valentinadx@hotmail.it (V.D.N.); maria.notarnicola@irccsdebellis.it (M.N.)

**Keywords:** colorectal cancer, bone tissue, growth factors, metastases

## Abstract

Colorectal cancer (CRC) is the third most common cancer worldwide. There is a need for the early diagnosis of CRC for a better prognostic outcome. It is, therefore, crucial to understand the CRC pathogenesis in all its aspects. In many cases, one of the main causes of cancer-related deaths is the presence of metastases. In this context, an often overlooked aspect is the metastatic tropism, since CRC, like other cancers, is more prone to metastasize some organs rather than others. Beyond the liver and lung, and differently from other types of cancers, a not usual site of CRC metastases is the bone. However, it may assume a crucial role in the development and the outcome of the disease. Therefore, this review aims to discuss the complex relations between bone markers and CRC pathogenesis, suggesting the use of these molecules as potential targets for therapeutic purposes. Different osteogenic molecules, some of whom are growth factors and are implicated in the different osteogenic pathways, have been proved to also be involved in CRC progression. Some of them are oncogenes, while others oncosuppressors, and in a future perspective, some of them may represent new potential CRC biomarkers.

## 1. Introduction

Colorectal cancer (CRC) is widespread across the world; it represents one of the most common cancers, and is among the leading causes of tumor death. Although the etiology of CRC relies on genetic causes, other factors (e.g., family history, inflammatory bowel disease, sex, smoking, folate intake, high intake of fats, alcohol, red and processed meats, sugars) can often actively contribute to its onset [1,2]. Besides, in 70% of CRC cases, it develops from previous neoplasms, such as colorectal bowel adenomatous polyps [3]. The ability of healthy colon epithelial cells to transform into neoplastic cells through the adenoma–carcinoma sequence has been largely described [4]. This sequence is regulated by oncogenes and oncosuppressors, subject to mutations and dysregulations that favor tumor development. High-performance techniques allow for quickly associating phenomena of genomic instability correlated with those processes promoting the development of cancer. These advances point to the possibility of early diagnosis and related therapeutic intervention [5].

A large part of the mortality rate from cancer, as well as tumor recidivism, is due to the staging and the presence or absence of metastases, where it has metastasized and whether there are micro- or macro-metastases. The process of the formation of metastases is characterized by several steps, all fundamental and essential to each other. These steps are not yet fully understood, as well as the molecular pathways that may regulate their occurrence, which genes are expressed and which are not. It is necessary that epithelial cells, following genetic mutations, become the trigger of what then evolves into carcinoma in situ. Later, some cancer cells detach from the primary mass to spread and place themselves in a distant site in the body where they form a metastasis [6]. This process is possible due to the occurrence of a process known as the “epithelial–mesenchymal transition” (EMT).

In this sequence of events, epithelial cells lose their different types of cell–cell and cell–extracellular matrix (ECM) junctions, and the apical–basal polarity, while acquiring an invasive, migratory capacity and secreting multiple components of the ECM [7]. Consequently, these cells are termed circulating tumor cells (CTCs), which invade the ECM, and enter the vascular system. Thanks to the blood flow, CTCs can reach a more distant site, where inputs of the “mesenchymal–epithelial transition” (MET) allow them to acquire capacities to perform extravasation and spread in the parenchyma. Following the inversion of the EMT process, cancer cells acquire epithelial properties again and, first of all, the high proliferative rate to create metastases [8].

Each tumor has preferential sites in which it produces metastases, the so called metastatic tropism [9]. Cancer formation and progression cannot be detached from cancer stem cells (CSCs). CSCs are fundamental in different aspects of tumorigenesis, such as tumor transformation, progression, therapeutic resistance and in metastatic tropism and, consequently, in the formation of metastases [10,11]. Therefore, a greater understanding of these mechanisms is crucial. The injection of allograft-derived pancreatic cancer tumor stem cells into wild type mice [9] demonstrated the production of metastases only in the liver or lung and liver, depending on whether the cell pool inoculation had been done by intrasplenic injection or in the caudal vein, respectively. It was also shown that the size of the metastatic masses is larger when they form in the liver than the lung. Overall, these findings support how metastatic tropism is affected by the presence of direct blood flow that, starting from the inoculation site, can reach distant organs. Following this event, the implantation of CSCs and the production of metastases is then influenced by the microenvironment of the host organ, which may be more or less suitable.

**Figure 1:**
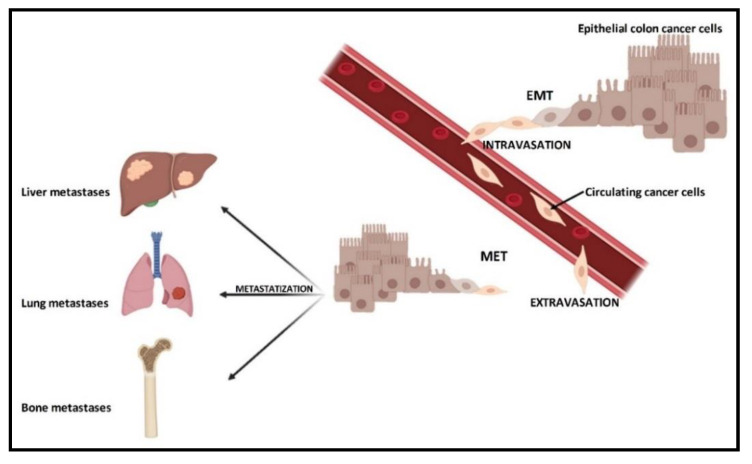
Colorectal cancer and its metastatic tropism. Primary tumor cells can be subjected to epithelial–mesenchymal transition (EMT), in order to generate mesenchymal cells with more motility and invasiveness. These mesenchymal cells enter the bloodstream, becoming circulating cancer cells (intravasation). Through the blood flow and under cellular signals, these cells reach distant sites where they metastasize. At this point, the circulating cancer cells come out from the blood stream (extravasation), undergo an inverse transformation, namely mesenchymal–epithelial transition (MET). Metastases are formed in preferential sites (metastatic tropism), such as liver, lung or bone.

As for CRC, after total surgical removal of the primary tumor mass, recurrences in the form of metastases can occur preferentially in the liver, lungs, lymph nodes, peritoneum and bone [12] (Figure 1). In this context, the colon and bone tissue, apparently so distant, have something in common. A disease in one of these apparatuses may well affect the physiological state of the other. They are more related than one can imagine. This review aims to describe what is known in the literature, reporting the state of the art on this topic.

## 2. CRC and Bone Metastases

Compared to liver and lung metastases, bone metastases in CRC occur only in 10–15% of cases [13]. In such patients, the five-year prognosis is less than 5% [14]. The diagnostic picture of these patients is very often characterized by skeletal-related events (SREs), which makes the clinical course of the disease worse. SREs can be constituted by the weakening of the bone structure, at both the trabecular and cortical level, and bone pain, as well as a higher probability of fractures. These pathological events worsen the patient’s survival and their quality of life [15,16]. In addition, gender and age are among the factors related to poor survival, Babu et al. presented a clinical study in which CRC patients with bone metastases were male and young. However, whether sex affects the prognosis of these subjects needs to be deeply investigated [17].

Santini et al. [18] collected the clinical data of a cohort of Italian CRC patients with different skeletal problems and bone metastases. According to their findings, the most affected bones by CRC metastases were the spine (65% of cases), hip/pelvis (34% of cases), long bones (26% of cases) and other bone sites (17% of cases). These percentages highlight the need for the early diagnosis of bone problems related to CRC and, therefore, for an equally early intervention to improve and extend the patient’s survival. To perform a timely early diagnosis of bone metastases, a scoring technique has been assessed using different clinical factors, such as tumor localization, lymph node metastases, and, finally, the presence of metachronous lung metastases as a third risk factor. This scoring technique can help clinicians immediately identify CRC patients most at risk for the development of bone metastases and make it possible to intervene directly with suitable therapies and relieve bone metastasis-related SREs [19,20].

Baek et al. [21] reported that only 1.1% of 5479 CRC patients showed CRC-related bone metastases. Most of these patients were at a late stage of cancer at the time of the CRC diagnosis. Bone metastases were already present at diagnosis in half of them, while the other half of the patients developed bone metastases during the course of the disease. As expected and independent from the presence of metastases in other organs, the presence of bone metastases is also associated with the presence of different SREs, and this situation led to painful patient survival.

In CRC, bone metastases usually develop later than those in other organs or tissue, such as liver and lung metastases, and there is a preferential link between bone and lung metastases. The prognosis is more severe in cases in which the metastasis of the tumor involves several sites simultaneously [22]. Bone metastases, perhaps more than others, are highly debilitating because of the various bone-related clinical pictures that they entail. Therefore, it would be helpful to be able to diagnose these metastases in a shorter time compared to their onset. Studies on this topic were performed by evaluating cases of rectal or colon cancer cases individually. Recently, Zhenghong et al. [22] have reported a higher percentage of bone metastases in rectal cancer patients than in CRC patients. Probably, this finding may depend on the broader vascularization in the rectum compared to the colon [23,24].

## 3. CRC and Bone Marrow

Several studies investigated the interactions between colorectal cancer and bone marrow (BM). Taketo et al. reported that the loss of the oncosuppressor SMAD4 is synonymous with CRC advancement. The authors noted, in both in vitro and in vivo experiments, that the loss of SMAD4 implies the lack of the block of expression of the gene C-C motif chemokine ligand 15 (CCL15) [25,26]. In this circumstance, CCL15 is expressed by cancer cells and induces the recruitment of CCR1+ myeloid cells from BM. The C-C chemokine receptor type 1 (CCR1) + cells have the characteristic of expressing and secreting matrix metalloproteinase 9 (MMP9), which is involved in tumor invasiveness by promoting tumor–stromal interactions. The analysis of human liver metastases, related to CRC, have shown that CCL15 expression, linked to a higher content of CCR1+ cells, is associated with a lower patient survival with respect to CCL15-negative liver metastases [26,27]. SMAD4 and CCL15 are inversely correlated, since the action of SMAD4 induces a negative regulation of the promoter of CCL15, causing the inhibition of CCL15 gene expression. Moreover, inhibitors of the CCL15–CCR1 axis have been suggested as potential therapeutic agents [26].

The role of BM-derived CCR1+ myeloid cells in CRC pathogenesis was also investigated by others research groups. In this regard, Kiyasu et al. very recently reported that the depletion of CCR1 induced a reduction in CRC growth. In particular, after reconstituting sub-lethally irradiated wild-type mice with the BM of wild-type or CCR1^−/−^ mice, they implanted colorectal cancer cells in these mouse models [28]. They noted that mice with CCR1 cell depletion showed a reduction in tumor growth and liver metastases, with respect to CRC mouse models with wild-type BM. The depletion of CCR1+ myeloid cells, genetically induced or by using an anti-CCR1 antibody, caused a suppression of CRC development, indicating CCR1 as a potential therapeutic target [28].

BM metastases, although rare, characterize CRC tumorigenesis due to their high vascularization. Furthermore, the formation of BM metastases is promoted by the slowness of the bone marrow bloodstream, which helps the deposition of the metastatic cells, and the presence of several growth factors, secreted following interactions between tumor cells and BM stroma [29]. The occurrence of these conditions creates the right conditions for tumor development. Metastases in BM often go unnoticed if they are mild, because they are not yet detectable with the most common imaging techniques, or they are detected late when they are well extended and cause severe pain or osteolytic fractures [29]. BM metastases are commonly observed in different solid tumors, such as breast, lung, prostate and, rarely, in CRC patients [30]. Chuwa et al. very recently described a case report of a CRC patient with BM metastases [31]. In particular, this patient presented disseminated carcinomatosis of bone marrow (DCBM). DCBM was diagnosticated by analysis of a BM biopsy, since the patient presented a persistent pancytopenia. BM biopsy analysis showed the infiltration of non-hematopoietic malignant cells and BM necrosis, pivotal features of DCBM [31]. The micro-metastasis of BM is related to poor prognosis [31,32].

An important role in CRC tumorigenesis is played by mesenchymal stem cells (MSCs). These cells, derived from BM, secrete growth factors, cytokines and chemokines into the stroma of developing tumors [33]. Nishikawa G. et al. reported that MSCs promote CRC progression through C-C chemokine receptor type 5 (CCR5) ligands, such as C-C motif chemokine ligand 3 (CCL3), CCL4 and CCL5. These ligands bind the receptor, CCR5, expressed by CRC cells [34]. The authors also observed that high serum levels of CCR5 ligands are related to a poor prognosis in CRC patients, therefore, CCR5 ligands could have value as predictive biomarkers. As previously reported by other groups, it was noted that an inhibition of CCR5, and consequently a reduction in the MSC–CRC cell interactions, corresponds to a reduction in tumor growth [34,35,36].

Although, to date, there are numerous studies indicating that CRC can present a bone-related symptomatology (i.e., osteolytic lesions, skeletal related events, etc.) due to bone metastases, it remains not fully clarified how CRC cells interact with bone cells. The result of this interaction is an imbalance between functional cells within the bone, i.e., osteoblasts and osteoclasts, usually in favor of the former, resulting in the formation of osteolytic metastases, due to a preeminent osteoclastogenesis [37]. In this process, chemokines play a relevant role, leading to the interaction between cancer and host cells. Different chemokines are implicated in the CRC cells’ chemoattraction to bone tissue, promoting cancer cell metastasis. The metastatic tropism is due to the interactions between ligands present on cancer cells and their specific receptors present on the cells of certain organs, or vice versa. Gong ZC et al. [38] very recently showed the relevance of CCL3, expressed by BM-derived monocytes, in osteoclastogenesis in CRC bone metastases. The authors reported that CRC cell-derived Epidermal Growth Factor (EGF) activates BM-derived monocytes and stimulates their high CCL3 expression. CCL3 promotes osteoclast maturation and, consequently, osteoclastogenesis [38]. Another interaction implicated in cancer cell recruitment has been demonstrated between CXCR4, expressed in CRC cells, and CXCL12, located in BM-derived cells. Furthermore, Itatani Y. et al. [12] described in detail other interactions existing between CRC cells and other myeloid cells.

Several proteins are differently involved in the relations between bone tissue and CRC by promoting tumor cell invasion and increasing the activity of other molecules with possible interferences in osteoinductive processes. A series of molecules, which are involved in these processes to varying degrees, is addressed below (Table 1).

## 4. Bone Morphogenetic Proteins (BMPs)

Although the interrelationship between bone tissue and a distant tumor can represent a common event, the association between bone and the CRC is rather rare. Clinical cases of heterotopic ossification have been reported in some tumors, and this process might also happen in some cases of CRC. The mechanism behind this pathological process is not well understood. However, immunohistochemical analyses revealed the involvement of specific proteins in bone tissue cells. The most accredited theory reports a mechanism similar to the EMT of mesenchymal cells in osteoprogenitor cells against molecular inputs secreted by cancer cells. These inputs consist of proteins secreted by cancer cells, bone morphogenetic proteins (BMPs). There are 24 different subtypes according to their amino acid sequences [39].

In the case of heterotopic ossification, the most common subtypes found include BMP2, BMP5, BMP6 and BMP9. The latter can be considered as one of the major osteoinductive factors [40]. All these proteins are essential for bone differentiation and maintaining the balance between tissue formation and erosion; osteoblasts, osteoclasts and chondrocytes produce them. BMPs are also implicated in the development of extraskeletal organs and neoplastic cells.

Thanks to molecular investigations on the colon that identified the expression of osteogenic proteins, Noh et al. [41] performed the analysis of a clinical case of heterotopic ossification in a CRC patient. The authors established that some cancer cells could express osteoblast phenotypical markers. These cells, with an osteoblast-like phenotype, secrete osteoinductive molecules, such as BMP9, osteocalcin and osteopontin. The release of these factors induces the transformation of other peritumoral cells, eventually leading to heterotopic ossification of the tissue.

### 4.1. BMP9

Bone morphogenetic protein 9 (BMP9) is one of the bone morphogenetic proteins. These molecules are part of the transforming growth factor-*β* (TGF-*β*) superfamily [42]. BMP9 and the others are implicated in various physiological processes, such as proliferation, migration, adhesion and apoptosis [42]. Therefore, their dysregulation can lead to the aberrant functionality of downstream signal pathways, eventually creating a protumoral context. This event occurs at the initiation of several neoplasms, also including CRC [43].

Among the others, BMP9 has been proved to be the one with the greatest osteoinductive effect [44]. BMP9 has two ways of action. The first is the canonical BMP/Smad pathway, which implies that, after the binding of BMP9 to its type I or II BMPR receptor, Smad 1/5/8 are activated by phosphorylation; then, Smad 1/5/8 bind Smad 4, and so this molecular complex moves to the nucleus where it activates other targets. The second path of action of BMP9 is the non-canonical BMP/Smad pathway, which involves the p38 MAPK and PIK3/AKT pathways [45,46].

The action of BMP9 is currently highly debated, as it seems that this protein does not show the same behavior in all tumors. BMP9 has been reported to have a protumoral action in liver sarcoma, ovarian sarcoma and osteosarcoma [47,48], while showing an anticancer activity in breast and gastric cancer [49,50]. As for CRC, Yuan et al. [51] evaluated the action of resveratrol both in vitro on LoVo cells and in vivo by inducing colon tumorigenesis in a mouse model. The findings from that study revealed that resveratrol acts through BMP9, which shows an antitumor and proapoptotic tendency. Furthermore, with the use of p38 inhibitors and the BMPR receptor, they demonstrated that the action of BMP9 in CRC is exerted by its binding to the receptor and by intracellular activation of the p38 MAPK pathway, also a fundamental pathway in osteogenesis induced by BMP9.

The involvement of BMP9 in colonic tumorigenesis has also been highlighted by an interesting and recent study on the potential beneficial anti-neoplastic effects of a natural component against CRC [52]. This study demonstrated that evodiamine (Evo), a quinolone alkaloid extracted from traditional herbal medicine *Evodia rutaecarpa*, had an antitumor activity, and the authors tried to elucidate its molecular mechanisms. By treating a colon cancer cell line, HCT116 cells, with Evo, an increase in BMP9 expression was observed. The authors evaluated whether the increase in the expression of BMP9 with the antitumor and anti-proliferative effects might involve hypoxia-inducible factor *α* (HIF-*α*). HIF-*α* is a proangiogenic factor, and it stimulates the formation of new vessels, an essential step for tumor progression. By treating HCT116 cells with Evo and using these cells to induce cancer in a mouse model following injection into the hip, the mechanism of the anticancer effect in CRC of Evo and BMP9 was investigated. In those experiments where BMP9 was overexpressed or silenced in HCT116, the antitumor action of Evo, its upregulation of HIF-*α* and its activation of the oncosuppressor p53 increased with BMP9 and was decreased by silencing BMP9 [52].

### 4.2. BMP5

Bone morphogenetic protein 5 (BMP5) also belongs to the BMP superfamily, a subgroup of the TGF*β* family. The homonymous gene encodes BMP5 as a pre-protein, which is enzymatically processed to give protein subunits arranged into homodimers and has a role in bone and cartilage development.

Like other BMPs, BMP5 is implicated in several malignancies, including CRC. Chen et al. [53] found a reduction in BMP5 expression both at gene and protein levels in CRC and that this lessening correlated with short patient survival. Furthermore, from sequencing genomic analyses, it has been clarified that, in some cases, these are loss-of-function type mutations. This evidence suggests that BMP5 has an anticancer action and, therefore, that its decline has a role in tumor transformation, as well as in EMT, and then in the loss of epithelial markers [54].

Studies of the transfection of colon carcinoma cells with a BMP5-expressing viral vector demonstrated that these cells overexpress a cell cycle regulator and tumor suppressor, cyclin-dependent kinase inhibitor 1C (CDKN1C), compared to cells in which BMP5 is down-expressed. CDKN1C induces a cell blockade in phase G1. Furthermore, the cellular overexpression of BMP5 also leads to a better expression of epithelial markers and a loss of mesenchymal markers [53].

With the advent of the microRNA era, the link between miR-32 and BMP5 has been better studied. The miRNA miR-32 is a short strand of RNA of only 20–25 nucleotides. The miRNAs negatively regulate gene expression and are involved in the regulation of physiological processes, but their dysregulation is often an explanation for pathological processes, including CRC.

The result of careful and profound bioinformatic analyses has highlighted the involvement of dozens of miRNAs in CRC patients. Among them, one of the most involved is miR-32 [55], which exhibits different functions depending on the pathology, behaving as either a tumor suppressor or oncogene. In CRC, it has been observed that dysregulated miR-32 acts with protumoral, pro-proliferative and invasive effects. Hence, miR-32 dysregulation is associated with poor patient survival. The authors identified BMP5 as a target of miR-32 by using expression and clinical data from The Cancer Genome Atlas (TCGA). They found that miR-32 expression levels are inversely correlated with BMP5 expression levels and that this correlation is stronger in advanced CRC [56].

## 5. Osteoprotegerin

Osteoprotegerin (OPG) belongs to the tumor necrosis factor receptor family (TNFR) and, as the name implies, is a soluble protein that protects bone tissue from the erosive action of osteoclasts. It is a protein secreted by osteoblasts. OPG is a decoy receptor for the receptor activator of nuclear factor kappa-B ligand (RANKL), a RANK receptor ligand; in this way, OPG evades the ligand-receptor bond RANKL-RANK, avoiding the maturation of osteoclasts, on whose surface RANK is present, and, therefore, their activation and erosion of the bone tissue. Hence, OPG acts as a negative regulator of bone turnover [57,58].

OPG is also expressed in several cancers, including CRC. In vitro studies show that colon cancer cells, such as HT-29 and SW480, express OPG both at the gene and protein levels. This result has been confirmed by in vivo studies in mouse models of colon tumors obtained after injection of the above-mentioned cell lines. In these models, the expression of OPG was found in tumors by using immunohistochemical analysis carried out on fixed and paraffin-embedded tumor masses [59,60]. Besides, the role of OPG was also investigated in the specific tumor context of CRC. OPG is also a decoy receptor for TNF-related apoptosis-inducing ligand (TRAIL), expressed by immune cells, and induces apoptosis in cancer cells. Therefore, the binding of OPG to TRAIL prevents its action, deflecting the problem of the TRAIL-induced apoptosis of cancer cells due to the binding of TRAIL to membrane death receptors, such as death receptors 4 and 5 (DR4-DR5) [60,61].

In vitro studies on colon carcinoma cells allowed us to observe this mode of action of OPG. In cells where OPG is silenced at the gene level by the use of siRNA or at the protein level, through the use of neutralizing antibodies in culture, TRAIL-induced apoptosis increases [59,62]. By using ELISA, high levels of OPG expression have been found in the culture medium of colon carcinoma cell lines.

Clinical trials have been conducted to measure serum OPG levels in patients with CRC. In these studies, OPG levels correlated with the staging of the tumor. Interestingly, the late stages of CRC corresponded to high serum OPG values [63,64]. Since the aggressiveness of CRC, as well as of all tumors in general, is also due to the resistance to apoptosis of cancer cells, one of the possible therapies is the exogenous administration of proapoptotic factors, such as TRAIL [65]. Tsukamoto et al. [66] demonstrated for the first time that gene and protein OPG expression increased in metastatic CRC cases compared to patients who have not yet developed metastases. However, CRC, especially when it is in an advanced stage, seems to be resistant to TRAIL, highlighting the problem of how to remedy this pathological condition. As a consequence, OPG is now considered as a potential prognostic and therapeutic target. The evaluation of its serum levels could allow for assessing the stage of pathology, as well as represent the object of targeted therapy [66].

The pro-tumor action of OPG in CRC was not confirmed by Kim HS et al. [67]. The authors reported that the OPG gene and protein expression levels decreased in colon cancer cells in comparison to normal colon epithelial cells. Besides, the downregulation of OPG was firstly hypothesized and then confirmed to be dependent on the degree of hypermethylation of the OPG gene promoter. When CRC cancer cells were treated with a demethylating substance, the OPG gene expression was recovered in comparison to the same cells before treatment. Therefore, OPG is described, rather, as an oncosuppressor in CRC, whose reduced expression may be considered as an index of the worsening of the clinical picture in CRC patients. According to the authors, these data, which contrast with previously published papers, may be explained by a comparison with normal colon epithelial cells, which has not yet been performed. Since the available clinical trials have not been performed on a consistent number of patients, there is a need for both further laboratory and clinical research. Based on the available data, OPG is undoubtedly actively involved in CRC tumorigenesis [67].

## 6. Osteopontin

Osteopontin (OPN) is a protein involved in bone remodeling and represents the major organic, non-collagenic component of the extracellular bone matrix. It is expressed by the precursors of osteoblasts and osteoclasts, as well as by mature osteoclasts, osteocytes and osteoblasts. As the name implies, OPN acts as a bridge between the osteoclasts and the inorganic part of the bone, the hydroxyapatite crystals, through the bond with *α*_v_*β*_3_ and CD44 receptors [68,69]. OPN is not only involved in bone turnover, but also in other physiological and pathological processes, including tumorigenesis.

OPN is a potential tumor marker in CRC. In fact, OPN gene and protein expression levels are higher in colon cancer cell lines than in normal colon epithelial cells, as well as in CRC tissue samples compared to non-tumor samples. The expression of OPN in CRC also correlates with the staging of the tumor, and, therefore, even with the survival of the patient, in whom high expression of OPN is synonymous with a poor prognosis [70].

The overexpression of OPN in colon cancer cells induces faster cell proliferation, with a higher migration capacity than cells transfected with the empty vector. These activities, promoting tumor progression, were confirmed in vivo by inoculating mouse models with OPN overexpressing cells. Under such conditions, larger tumor masses and metastases may develop compared to controls. Immunohistochemical analyses by CD31 antibodies have shown higher angiogenesis in OPN overexpressing tumor masses than those obtained by inoculating colon cancer cells transfected with an empty vector in mouse models. Overall, these results support OPN as a CRC tumorigenesis factor that increases the metastatic potential of the tumor [71].

The mechanism underlying the involvement of OPN in tumorigenesis has been clarified by using a murine cell line of colon cancer, CT-26 cells, which are highly invasive, have metastatic potential and express OPN at high levels. CT26 cells silenced for OPN were inoculated in immunosuppressed mouse models. Cells with silenced OPN showed a lessened formation of metastases compared to control cells, due to the reduction in motility and cell invasiveness. The reduced cell invasiveness is explained by a decreased expression of matrix metalloproteases, specifically of MMP-2 and MMP-9 [72,73]. These data were also confirmed by experiments with siRNA-OPN. Gene silencing of OPN reduced the proliferation, adhesion and migration of another human colon carcinoma, LoVo cells. Besides, these results were associated with a lessening of the angiogenic process in CRC [74].

Gene analyses have made it possible to identify recurrent single nucleotide polymorphisms (SNPs) associated with a greater tendency for tumor development. Data analysis on Chinese CRC patients revealed a higher risk of developing CRC in the presence of the A allele in the SNP rs9138 in the 3’UTR region of the OPN gene and allele C in the SNP rs1126616 in exon 7 of the OPN gene. However, no statistically significant relationship between the patient’s genotype and serum OPN levels were found [75]. On the contrary, Kamal et al. [76] found contrasting data by carrying out the study on Egyptians. This finding is not so surprising since different ethnic groups may show varied polymorphisms, and the same allele in a SNP can give different results in diverse populations.

The aggressiveness and invasiveness properties of cancer cells correlate with a lower state of differentiation and with stem characteristics. The association between the protumor properties of OPN in CRC and cancer cell stemness was found by Ng et al. [77]. After the induction of the overexpression of OPN in the DLD-1 cell line, the authors reported that these cells show high levels of expression of some stem cell markers, SOX2 and OCT4. Furthermore, by treating these DLD1-OPN cells with oxaliplatin, the cells showed resistance to apoptosis compared to the control DLD-1 cells. These data were then confirmed in vivo in CRC tissues and patients. A positivity for SOX2 expression was found in OPN-positive CRC tissues, demonstrating that cancer cells show stem properties. Oxaliplatin-treated CRC patients, who were resistant to the chemotherapy drug, showed higher OPN expression levels than drug-sensitive CRC patients. Therefore, OPN could even be a reference value for identifying a CRC patient’s response to oxaliplatin treatment [77].

Lastly, the different possible mechanisms by which OPN promotes tumorigenesis in CRC include autophagy, which appears to be dysregulated in this neoplasm, and through alterations in the p38-MAPK pathway [78]. OPN can inhibit the autophagy process through the p38-MAPK pathway, as demonstrated by an in vitro study on the HCT116 cell line. The administration of exogenous OPN to these cells induced augmented p38-MAPK phosphorylation, along with a reduction in the gene and protein expression of molecules involved in the formation of the autophagosome (such as Beclin1, Atg4b, Bnip3 and Vps34) [79].

## 7. Other Proteins

### 7.1. Bone Sialoprotein

Bone sialoprotein (BSP) is the structural glycoprotein of the bone matrix that defines the initial process of bone formation and constitutes 15% of the non-collagen proteins of this tissue. It is expressed by different cells of the bone tissue and the cartilage. Like OPN, BSP has an important domain for binding with *α*v*β*3 integrin, which mediates the interaction of osteoblasts and osteoclasts with the bone matrix during bone resorption. In particular, it has been shown that BSP regulates the processes of deposition and mineralization of the bone, but it is also involved in the opposite process, bone resorption [80]. Some findings also suggest the expression of BSP in various neoplasms and non-tumoral diseases. A study by Fedarko et al. [81] described the expression of BSP in the serum of patients with different cancers, including CRC samples, and reported that circulating BSP levels were significantly elevated in CRC patients.

### 7.2. Tartrate-Resistant Acid Phosphatase

Tartrate-resistant acid phosphatase (TRAP) is a glycosylated metalloprotein and has a fundamental role in many biological processes of skeleton development. TRAP is highly expressed in osteoclasts [82]. It is capable of degrading phosphoproteins of the bone matrix, including OPN and BSP. OPN and BSP, even when partially dephosphorylated, are unable to bind osteoclasts. Therefore, it has been hypothesized that TRAP is secreted by the osteoclast, dephosphorylates osteopontin and thus allows osteoclast migration and the occurrence of further resorption [83]. In addition to these functions, TRAP, also being expressed by activated macrophages, seems to have a role in innate immunity. A study performed on a cohort of CRC patients by Nagorsen et al. [84] found that the high expression of TRAP is associated with longer survival in CRC patients and a reduction in mortality. This finding was independent from age, since both young and older patients showed comparable percentages. There are two subclasses of macrophages, the anticancer M1 and the protumoral M2. Although, in some pathological contexts, these two subclasses and their respective roles are clear, in CRC, there are controversial opinions. In fact, in addition to the known role of M1 macrophages, an improvement in prognosis in CRC is also reported following the in situ recruitment of M2 macrophages.

How et al. [85] reported an analysis of which subclass expresses TRAP in CRC by using immunohistochemical analyses that made it possible to achieve the goal by double marking the CRC tissue samples. The double positivity to TRAP and CD68, a macrophage marker, revealed that macrophages express TRAP. Furthermore, the double positivity to TRAP and CD163, a marker of the M2 subclass, has shown that both M2 and non-M2 macrophages express TRAP. These results provide evidence that TRAP macrophage expression is associated with improved prognosis and implies that TRAP is a potential biomarker in CRC.

### 7.3. Runt-Related Transcription Factor 2

Runt-related transcription factor 2 (Runx2), belonging to the RUNX heterodimeric transcription factor family, was the first osteoblast-specific transcription factor to be identified and is still considered the master gene for osteoblastogenesis. KO mice for the RUNX2 gene have a cartilaginous skeleton and the complete absence of osteoblasts, irrefutably demonstrating the role of RUNX2 in osteoblastic differentiation [86]. Several pieces of evidence report the role of RUNX2 in tumorigenesis in various organs. As for CRC, Sase et al. [87] assessed the expression levels of RUNX2 in CRC tissue samples, reporting their positivity compared to normal epithelial cells and that this result was related to poor patient prognosis. Besides, from in vitro analyses of colon cancer cells, i.e., SW480 and DLD1, RUNX2 gene silencing experiments showed a reduction in the proliferation, migration and invasion of cells compared to control cells. Previous studies evaluated the association between the ER*β* estrogen receptor and RUNX2 [88], and based on them, the authors have conducted further experiments to clarify the concept. By blocking the expression of ER*β* by gene silencing or with a protein-level inhibitor, a reduction in RUNX2 levels in vitro, as well as a reduction in the proliferation of these colon cancer cells, were shown. These results make it clear that RUNX2 is an important prognostic factor for CRC and seems to be regulated, at least partially, by ER*β* [87].

### 7.4. Trasforming Growth Factor β1

During the resorption process, many factors are released from the bone matrix that direct the migration of mesenchymal stem cells (MSCs) to the newly eroded surface. Among these factors, there is transforming growth factor (TGF)-*β*1, one of the most abundant cytokines in the matrix. TGF-*β*1 is a cytokine involved in the regulation of numerous cell functions, ranging from growth to apoptosis. It has been shown that this factor can regulate the proliferation and differentiation of osteoprogenitor cells [89], but the possible action of TGF-*β*1 was also evaluated in colon cancer cells [90]. TGF-*β*1 inhibits the proliferation of HT-29, cells that show resistance to chemotherapy treatment-induced apoptosis. Besides, TGF-*β*1 weakens the expression of cyclooxygenase 2 (COX-2), a well-known inflammation-promoting enzyme that is adapted to a tumor context. This result is reversed by treating HT-29 with an ERK inhibitor, confirming that ERK mediates the effect of TGF-*β*1 on COX-2. To further clarify this mechanism, the authors assessed whether ERK activation was direct or indirect, evaluating the involvement of ROS. HT-29 cells were treated with both TGF-*β*1 and an antioxidant substance. The findings from the study highlighted a loss of the activation of ERK by TGF-*β*1, suggesting that TGF-*β*1 is involved in the inhibition of tumor growth in HT-29, the induction of apoptosis by ROS production and that this event is upstream of ERK activation [90].

## 8. Conclusions

Colorectal cancer metastasis is a complex process with many molecular components that act as oncogenes and oncosuppressors. Numerous clinic studies have clarified that bone biomarkers are important players in CRC, as some of them correlate with cancer development and prognosis.

In this review, we pointed out that numerous osteogenic molecules regulate many pathological aspects of CRC, including the initiation of inflammation, tumoral cell transformation, metastatic capacity acquisition, epithelial–mesenchymal transition, intravasation, extravasation, mesenchymal–epithelial transition and metastasis formation.

After elucidating the molecular mechanisms that support the bone biomarkers’ actions in CRC pathogenesis, new bone molecule-based therapies may be realized. Interestingly, the manipulation of endogenous bone biomarkers by administering siRNA inhibitors could be useful in modulating the expression of downstream pathways. To date, no therapies targeting these molecules have been developed to treat CRC in human clinical trials. Despite this, the use of these bone molecular factors as therapeutic targets is very promising since they are able to regulate the course of the neoplasm.

In conclusion, although the intestinal tract and bone tissue seem to be so far from each other in terms of anatomy, embryology and physiology, they are more related than one can imagine. Several relations have been demonstrated between these two organs, implicating different molecules. The study of their molecular relations opens new horizons for diagnosis and therapies for CRC patients.

## Figures and Tables

**Table 1:** Molecular factors and their mechanisms of action in bone tissue and in Colorectal Cancer (CRC).

Molecular Factor	Mechanism of Action in Bone Tissue	Mechanism of Action in CRC	References
BMP9	Stimulation of the production of bone tissue	Antitumoral, pro-apoptotic	[26,27,28,29,30,31,32,33,34,35,36]
BMP5	Stimulation of the production of bone tissue	Antitumoral, pro-apoptotic	[37,38,39,40]
OPG	Protection of bone tissue from the erosive action of osteoclasts	Oncogene/oncosuppressor	[41,42,43,44,45,46,47,48,49,50,51]
OPN	Involvement in bone remodeling/bone turnover	Promotion of tumorigenesis	[52,53,54,55,56,57,58,59,60,61,62,63]
BSP	Involvement in both bone formation and bone erosion	Protumoral biomarker	[64,65]
TRAP	Osteoclast maturation, bone erosion	Antitumoral biomarker	[66,67,68,69]
RUNX2	Involvement in osteoblastogenesis	Protumoral, anti-apoptotic	[70,71,72]
TGF*β*1	Regulation of the proliferation and differentiation of osteoprogenitor cells	Antitumoral	[73,74]
